# The population impact of obesity, sedentary lifestyle, and tobacco and alcohol consumption on the prevalence of type 2 diabetes: Analysis of a health population survey in Chile, 2010

**DOI:** 10.1371/journal.pone.0178092

**Published:** 2017-05-25

**Authors:** María P. Bertoglia, Juan G. Gormaz, Matías Libuy, Dérgica Sanhueza, Abraham Gajardo, Andrea Srur, Magdalena Wallbaum, Marcia Erazo

**Affiliations:** 1 Public Health Nutrition Program, School of Public Health, University of Chile, Santiago, Chile; 2 Molecular and Clinical Pharmacology Program, Bio-Medical Sciences Institute, University of Chile, Santiago, Chile; 3 Noncommunicable Diseases Department. Ministry of Health, Santiago, Chile; Mexican Social Security Institute, MEXICO

## Abstract

**Aim:**

To estimate the impact of tobacco use, sedentary lifestyle, obesity and alcohol consumption on type 2 diabetes mellitus (T2DM) prevalence in the Chilean population.

**Methods:**

The study-included 5,293 subjects with fasting glycaemia levels from the nationwide cross-sectional health survey in 2010, commissioned by the Ministry of Health, Chile. Crude and Adjusted Odds Ratio to T2DM and its corresponding 95% confidence interval were estimated through logistic regressions. Attributable fractions and population attributable fractions were estimated.

**Results:**

T2DM prevalence was 9.5%. Sedentary lifestyles and obesity were significant risk factors for T2DM. 52,4% of T2DM could be avoided if these individuals were not obese, and at a population level, 23% of T2DM could be preventable if obesity did not exist. A 64% of T2DM is explained by sedentariness, and if people would become active, a 62,2% of the cases of diabetes could be avoided.

**Interpretation:**

About 79% of T2DM cases in Chile could be prevented with cost-effective strategies focused on preventing sedentary lifestyle and obesity. It’s therefore urgent to implement evidence-based public health polices, aimed to decrease the prevalence of T2DM, by controlling its risk factors and consequently, reducing the complications from T2DM.

## Introduction

Noncommunicable diseases are the principal cause of mortality worldwide. Diabetes by itself represents 4% of deaths, and has been identified as the leading cause of disability[1]. Recently, it was estimated that about 347 million adults have diabetes worldwide, a condition that is rising in different regions of the world, being led by Asia and Africa[2].

The World Health Organization has declared that unhealthy diets, sedentary lifestyles, tobacco and excessive alcohol consumption are the major risk factors for type 2 diabetes mellitus (T2DM)[1].

Obesity increases the prevalence of diabetes, presenting a dose-response relationship with Body Mass Index with no sex difference[3]. Physical inactivity is another main risk factor for T2DM, causing 7% of the burden of disease from T2DM worldwide[4]. Alcohol consumption exhibits a U-shaped relationship with the risk of T2DM in both males and females, with two drinks per day (~50g/day) increasing the Relative Risks (RR)[5]. Recently, tobacco consumption has been pointed out as having a causal association with T2DM. A recent meta-analysis that included 21cohort studies reinforced that smoking is an independent risk factor for T2DM. The pooled relative risk (RRc) is 1.44 (95% CI = 1.31 to 1.58), showing that there’s a dose-response relationship[6]. Also, among the key conclusions of the 2014 Surgeon General’s report, research continues to identify new diseases caused by smoking, including T2DM [7].

In Chile, T2DM has increased over the last years. In year 2015, diabetes national prevalence in subjects aged 20 to 79 was 11% [uncertainty range 9.3–13.6%], which translates in a total of 1,37 [1,16–1,69] million people living with diabetes.

This locates Chile among the top 5 countries with the highest prevalence of T2DM within the Central and South American Region[8].

In order to implement public policies that reflect the epidemiology of this disease, it’s necessary to estimate the risks and the population impact of these four behavioral risk factors (tobacco use, sedentary lifestyles, obesity and excessive alcohol consumption) on T2DM prevalence. Thus, the aim of this study is to estimate the impact of these four risk factors in the Chilean population and their prevalence in patients suffering T2DM. For these analyses, the 2009–2010 National Health Survey data will be used (prevalence, measure of association and burden of disease).

## Materials and methods

### Study design

The Chilean Ministry of Health (MINSAL) has conducted two nationwide cross-sectional Health Surveys (NHS). The first NHS was completed in 2003, and included the screening of people 17 years and older. These people were recruited by using a stratified random sample representing the adult population, considering their socioeconomic status, urban/rural residence and educational level[9].

The survey conducted during 2009–2010 was designed to follow up some of the health problems included in the NHS 2003, but also incorporated new diseases, conditions or chronic health problems, risk factors and issues related to perceived health status in the sampled population. A total of 13 conditions previously evaluated in 2003 (high blood pressure, dyslipidaemia, nutritional status, diabetes mellitus, smoking, metabolic syndrome, cardiovascular risk, sedentary lifestyles, musculoskeletal symptoms, renal function, chronic respiratory symptoms, cognitive impairment of the elderly and B and C hepatitis virus) were also evaluated in 2010[10]. This second Health Survey used a complex sampling representative of the Chilean population (15 years and older) and is the data used in this publication to measure population impact. (http://epi.minsal.cl/bases-de-datos/)

### Sampling and sample size

A total of 5,293 individuals were included in the analysis with more than 8 hours of fasting to measure their glycemia levels.

The sampling frame was build from the 2002 Population and Housing Census. This cross-sectional study used a complex sample design (multistage stratified cluster sample of households) with national, regional and rural/urban area representation. The target population was adults aged older than or equal to 15 years old. The survey had a response rate of 85%, the rejection rate was 12% (n 391), and 632 subjects were excluded because physical examination was not performed. Thus, 5,293 individuals were finally interviewed. Nurses performed clinical measurements and tests to 5,043 participants and 4,956 people accepted laboratory tests (blood and urine samples). The sample loss was 28% and included refusal, inability to contact and other random loss.

The sample was designed with over-representation of the elderly and people living in areas other than the Metropolitan Region (capital region), including individuals living in rural areas, in order to increase the sample design efficiency and homogenizing the precision of the estimators.

Pregnant women and persons with violent behaviour were excluded from the random selection.

### Laboratory analysis

Specialists from the ‘Catholic University of Chile’ conducted all laboratory analysis and interpretation of clinical tests. Once the survey was finished, the test results were sent to each participant, along with health recommendations according to their results. In addition, local epidemiologists provided information to each participant according to their risk level. The survey incorporated quality control processes at different stages, including a National Training Program for interviewers, an interviewer manual and the use of electronic devices to obtain automated information. These provided an interim standardization in addition to high quality laboratory analysis techniques to ensure essential methodological survey procedures.

### Data processing and statistical analysis

The Ministry of Health provided a complete database with the cases and variables incorporated in the survey. Data consistency was reviewed through the analysis of determined variables distribution.

Shapiro-Wilk test was used to determine if the variables presented Gaussian distribution. Statistical tests used to evaluate differences among groups depended on the variable distribution (parametric and nonparametric tests).

Accordingly to the health-related definitions used by the National Health Survey, the variables incorporated in this article are:

- T2DM (if any of these two criteria were met, a respondent was deemed to have T2DM):
  - T2DM self-report (not during pregnancy).
  - Fasting glycaemia ≥126 mg/dL.
- Smoking status (self-reported):
  - non-smoker (never smoked),
  - former smoker (≥ 6 months of quitting tobacco)
  - and current smoker (daily and occasional smoker, and < 6 months of quitting tobacco smoke).
- Obesity: Individuals with a Body Mass Index ≥ 30 Kg/M^2^.
- Excessive alcohol consumption: Determined by using the Abnormal Alcohol Consumption Scale ≥ 2 points[11]. This Scale has been validated in Chile and it’s widely used in the country (this cutting point is referred to abnormal alcohol consumption at some time in life)[10].
- Sedentary lifestyle: Less than 30 minutes of physical activity, a minimum of 3 times a week.

Crude and Adjusted (for the risk factors sedentary lifestyle, obesity and alcohol consumption) Odds Ratio to T2DM and its corresponding 95% confidence interval (95% CI) were estimated through logistic regressions. Attributable fractions (AF) are interpreted as the proportion of disease risk that could be prevented if the exposure was eliminated. This has a practical value in public health prevention policies, especially when the exposure is modifiable[12]. It was calculated using the following formula: AF = P(D)-(D|Ē)/P(D), where P(D) is the probability of disease, and P(D|Ē) is the probability of disease not exposed to the risk factor under evaluation. Population attributable fractions (PAF) are calculated using Levin’s formula[13].

It’s important to acknowledge some considerations about the use of these risk measures in cross-sectional studies. In first place, the risk measure is calculated from the odds ratio rather than from the risk ratio[14]. Also, as the analysis rely on prevalence data to study the association between disease and a risk factor, the following minimal assumptions are fulfilled[15]: T2DM does not lead to a change in smoking habits; smoking is not a prognostic factor of the outcome (T2DM duration is independent of the smoking status); and smoking status information is relevant for the T2DM (adequate time frame).

The analysis was performed using STATA Software, version 12[16].

### Ethical aspects

The Catholic University of Chile School of Medicine Research Ethics Committee granted its ethical approval. Patients were invited to participate in the study. Before their incorporation, the objectives of the study, measurements and risks were informed to each patient, if they agreed with the protocol, an informed consent were signed for each participant.

## Results

The population’s characteristics are shown in Table 1.

**Table 1:** NH survey of population characteristics.

Characteristic	2010		
	Total (n = 5293)	Males (n = 2150)	Females (n = 3143)
Age (Mean ± SD)	46.37 ± 18.68	45.62 ± 18.55	46.87 ± 18.75
Education (%) < 8 years 8–12 years > 12 years	26.70 54.67 18.62	24.44 56.48 19.08	28.25 53.44 18.31
Diabetes Mellitus %	9.5	8.4	10.4
Alcohol intake (%) (Problem Drinker)	14.72	27.62	6.04
Sedentary lifestyle (%)	91.66	88.11	94.09
Obesity (BMI ≥30 Kg/m^2^) (%)	29.1	23.36	32.97
Smoking (%) Current Former (>6 months)	40 19.36	39.28 23.88	33 16.29

The risk factors for T2DM included in this study were identified as obesity 29.1%, sedentary lifestyle 91.66%, lifetime smokers 40%, former smokers 19.36%, and excessive alcohol consumption (problem drinker) 14.72%.

When OR were estimated, sedentary lifestyle, obesity and alcohol consumption became significant in crude analysis, and when adjusted, only obesity and sedentary lifestyle remained significant (Table 2).

**Table 2:** Crude and adjusted odds ratios and 95% confidence interval for tobacco, obesity, sedentary lifestyle, excessive alcohol consumption and diabetes mellitus.

Exposure	Total		Males		Females	
	2010 (n = 5293)		2010 (n = 2150)		2010 (n = 3143)	
	Crude	Adjusted*	Crude	Adjusted*	Crude	Adjusted*
Obesity	2.37 (1.97–2.85)	2.20 (1.82–2.66)	2.18 (1.60–2.96)	1.93 (1.40–2.66)	2.48 (1.96–3.13)	2.35 (1.85–2.99)
Sedentary	2.890 (1.81–4.61)	2.248 (1.38–3.65)	3.272 (1.65–6.46)	2.604 (1.30–5.19)	2.405 (1.25–4.59)	1.878 (0.94–3.74)
Alcohol consumption	0.67 (0.50–0.88)	0.84 (0.62–1.14)	0.65 (0.45–0.93)	0.80 (0.55–1.18)	0.78 (0.47–1.31)	0.91 (0.52–1.59)
Former smoker (>6 months)	1.16 (0.93–1.44)	1.14 (0.90–1.44)	1.71 (1.21–2.41)	1.57 (1.10–2.26)	0.90 (0.66–1.22)	0.86 (0.62–1.19)

*Adjusted for the other risk factors.

When stratified by sex, in crude analysis, the four factors were significant for men (alcohol is a protective factor however), and when adjusted; only obesity, sedentary lifestyle and former smoker remained as significant risk factors while alcohol consumption was not statistically significant. For women, only obesity and sedentary lifestyle were significant and when adjusted, only obesity remained as a risk factor for T2DM (Table 2).

When attributable fractions were estimated, sedentary lifestyle was the main risk factor that explains T2DM with an overall value of 55–58, followed by obesity (with values ranged between 52 and 55), and former smokers (between 12 and 38).

When stratified by sex, in males, the principal risk factor is sedentary lifestyle (61.54) compared to women, to whom obesity is the main risk factor explaining T2DM (57.45). Being a former smoker is significant in men (Table 3).

**Table 3:** Adjusted attributable fractions (AF), population attributable fractions (PAF) and 95% confidence interval for tobacco, obesity, sedentary lifestyle, excessive alcohol consumption and diabetes mellitus.

Exposure	Estimator	Total	Males	Females
		2010	2010	2010
Obesity	AF	54.55 (53.65 to 55.44)	48.19 (47.08 to 49.30)	57.45 (56.41 to 58.48)
	PAF	25.26 (18.72 to 31.27)	18.26 (8.16 to 27.25)	29.52 (20.81 to 37.28)
Sedentary	AF	55.36 (53.90 to 56.81)	61.54 (59.66 to 64.46)	46.52 (44.93 to 48.12)
	PAF	53.55 (26.0 to 70.84)	58.69 (20.47 to 78.54)	45.44 (-6.1 to 71.93)
Alcohol consumption	AF	-19.05 (-19.75 to -18.34)	-23.46 (-24.24 to -22.67)	-8.70 (-9.68 to -7.72)
	PAF	-1.98 (-5.44 to 1.35)	-4.96 (-13.96 to 3.32)	-0.43 (-3.06 to 2.12)
Former smoker (>6 months)	AF	12.28 (11.55 to 13.01)	36.31 (35.25 to 37.36)	-16.28 (-17.01 to -15.55)
	PAF	3.28 (-2.6 to 8.84)	14.85 (2.57 to 25.58)	-2.77 (-9.02 to 3.11)

Finally, when the impact of these risk factors on the population is considered, about 82% of the T2DM prevalence rate in the country is explained by sedentary lifestyle, obesity and tobacco. The major impact is given by sedentary lifestyle, explaining ~54% of T2DM at a national level, being more important in men than in women (~59 vs. ~45, respectively), followed by obesity with ~25% of the cases in the country (~30% in women and ~18% in men). Being a former smoker (~3% overall) was found to be only significant in men (~15%). All of these figures are displayed in Table 4.

**Table 4:** Population impact of sedentary lifestyle, obesity and tobacco on diabetes mellitus prevalence.

Population	Total †	T2DM Prevalence	Population with T2DM	PAF to obesity	PAF to sedentary lifestyle	PAF to smoking (former > 6 months)	Population with T2DM attributable to obesity	Population with T2DM attributable to sedentary lifestyle	Population with T2DM attributable to tobacco	Total
Men	7668740	8.4	644174	18.26	58.69	14.85	117626	378065	95660	591351
Women	7447695	10.4	774560	29.52	45.44 **	-2.77 **	228650	351960	-21455	559155
Total	15116435	9.5	1436061	25.26	53.55	3.28 **	362749	769011	47103	1178863

^†^ National Census 2002

**Not significant

## Discussion

This article illustrates that three of the four risk factors considered in the analysis have an impact on the prevalence of type 2 diabetes mellitus. They are: sedentary lifestyle, obesity and tobacco consumption (tobacco demonstrated to be a risk factor only in men). Physical inactivity and obesity have the highest population impact on this disease.

### Similar studies

Similar studies using the same methods to evaluate the association between risk factors and diabetes found no significant evidence of alcohol being one of the risk factors for type 2 diabetes, furthermore, moderate alcohol consumption has been described as protective for T2DM[5]. The proposed explanation of this protection is that moderate alcohol consumption protects cardiovascular health, especially in men when consuming 22g/per day of alcohol (RR 0.87 CI 0.76 to 1.00). Meanwhile in women, consumption of 24 g/per day of alcohol is even more protective (0.60 CI 0.52 to 0.69), when compared to lifetime abstainers[5].

Based on an analysis of BMI and overall mortality, a collaborative study notes that, in both male and female, mortality was lower at a BMI between 22.5–25 kg/m^2^[3]. Above this range, positive associations for different specific causes of mortality were recorded. The absolute excess risk attributable to BMI and smoking is additive, i.e., for each increment of 5 kg/m^2^ units of BMI there is an average increase of 30% in overall mortality (hazard ratio per 5 kg/m^2^: 1, 29 (95% CI 1.27 to 1.32)), 40% for cardiovascular related mortality (HR 1.41 CI 1.37 to 1.45), 60–120% for diabetic cause of mortality (HRs 2.16 CI 1.89–2.46), kidney (1.59 CI 1.27 to 1.99) and liver (1.82 CI 1.59 to 2.09), 10% for mortality from cancer (HR 1.10 CI 1.06 to 1.15), 20% for respiratory causes (HR 1.20 CI 1.07 to 1.34) and 20% to other causes of mortality (1.20 CI 1.16 to 1.25)[3].

A recent study estimated the population attributable fraction of physical inactivity. Life tables were used to estimate the gain in life expectancy of the population. Globally, it was estimated that physical inactivity causes 6% (range 3.2% in East Asia to 7.8% in Eastern Mediterranean) of the burden of disease related to coronary heart disease, 7% (3.9–9.6%) of T2DM, 10% (5.6 to 14.1) of breast cancer and 10% (5.7 to 13.8) of colon cancer. It was estimated that avoiding physical inactivity could increase the world’s population life expectancy in 0.68 years (range 0.41 to 0.95)[4].

### Biological basis

Over the past decade, several data showed the role of regular exercise in the prevention of T2DM, as well as it’s beneficial effects on glycemic homeostasis [17]. Recent evidence showed a reduced exercise capacity in patients with T2DM compared with non-diabetic individuals[18]. This phenomenon can be explained because insulin stimulates the muscle’s glucose uptake, which is responsible for disposing 80% to 90% of the consumed glucose load[19]. The resultant hyperglycaemia presents a stimulus to the beta cells, which secretes large amounts of insulin after meals, and it’s directly involved in the generation of insulin resistance and diabetes[20]. From a biochemical standpoint, evidence has shown that intramuscular nonoptimal lipid metabolism provide the substrate to metabolite formation associated with the development of insulin resistance through different pathways[21]. In this context, it has been proposed that sedentarism is associated with mitochondrial dysfunction in the skeletal muscle [22].

Several evidence showed an association between smoking and insulin resistance[23,24], the sub clinical condition prior to the development of T2DM [25]. In 1992 Facchini et al.[26], studied the effects of smoking over glycemic homeostasis, comparing the response of 20 healthy individuals (mean age of 39 years old) with a smoker group. The smoker group had significant higher insulinaemia. After that, Attval et al.[27], compared the peripheral insulin sensibility when smoking, finding that peripheral glucose uptake decreases.

The increase in Insulinaemia would be mediated mainly by the ability of nicotine to induce a chronic increment of insulin antagonist in plasma, such as catecholamines, cortisol and growth hormone (GH)[28–32]. An increase of catecholamines reduces the peripheral sensitivity to insulin [33,34] and the secretion of this hormone[35]. Chronic plasmatic cortisol elevation has been associated with insulin resistance, independently of obesity[36] through a mechanism that would impair β-pancreatic function and peripheral tissue insulin sensitivity[37]. Growth hormone increase induces insulin resistance through altered hepatic metabolism, decreasing peripheral recruitment of glucose and β-pancreatic effects[38].

Several epidemiological studies indicate that central obesity is an important risk factor for T2DM[39]. Adipose tissue is an active secretory organ whose secretion profile drastically changes with overweight and obesity, increasing the circulating concentrations of adipokines like leptin, or resistin [21]. Furthermore, adipose tissue macrophages starts to secrete inflammatory cytokines such as TNF α[40]. An increase in circulating levels of these adipocyte- and macrophage-derived factors in obesity leads to a chronic low-grade inflammatory state that has been linked to the development of insulin resistance and T2DM[22].

On the other hand, Obesity-related accumulation of ectopic fat in key insulin-sensitive organs (e.g., skeletal muscle and viscera) causes changes in the insulin-signaling pathways[41]. Liver steatosis is an important trigger of insulin resistance and pre-diabetes, suggesting that accumulation of intrahepatic fat is more harmful than the accumulation of ectopic fat elsewhere in the body[42]. A study[43] reported that accumulation of liver fat might affect β-cell compensation for insulin resistance.

Despite that epidemiological data found that alcohol consumption reduces the incidence of T2DM, literature also suggest that binge drinking seems to increase the incidence of this disease[44]. However, these findings seems not to be applicable to some populations in Asia, where available data suggest that alcohol intake may be a risk factor for T2DM mellitus for Japanese[45]. Chronic ethanol consumption may produce steatohepatitis [46], promoting the development of T2DM through a liver dependent pathway.

### Study limitations

A group of people did not accept to provide blood samples, which were necessary to detect T2DM patients without self-report, according to the study definitions: therefore, it’s possible that T2DM prevalence has been underestimated.

Finally, as in all cross-sectional study, it’s not possible to affirm a causal association between T2DM and the researched risk factors because all of them were measured at the same time. However, our findings could help estimate at a population level the impact of public health policies to prevent T2DM. In Chile’s case, where NCDs and their risk factors are on the rise and a high impact of these risk factors have been observed on diabetes, it’s urgent to implement interventions to tackle them.
